# Discovering biomarkers associated with infiltration of CD8^+^ T cells and tumor-associated fibrosis in colon adenocarcinoma using single-cell RNA sequencing and gene co-expression network

**DOI:** 10.3389/fimmu.2025.1496640

**Published:** 2025-03-31

**Authors:** Jinning Zhang, Ziquan Sun, Guodong Li, Lixian Ding, Zitong Wang, Ming Liu

**Affiliations:** ^1^ Colorectal Cancer Surgery Department, The Second Affiliated Hospital of Harbin Medical University, Harbin, Heilongjiang, China; ^2^ Future Medical Laboratory, The Second Affiliated Hospital of Harbin Medical University, Harbin, Heilongjiang, China; ^3^ Department of General Surgery, The Fourth Affiliated Hospital of Harbin Medical University, Harbin, Heilongjiang, China; ^4^ Heilongjiang Province Key Laboratory of Digestive Surgery and Nutrition & Metabolism, The Fourth Affiliated Hospital of Harbin Medical University, Harbin, Heilongjiang, China

**Keywords:** colon adenocarcinoma, CD8 + T cells, single-cell RNA sequencing, fibrosis, prognostic biomarkers

## Abstract

**Background:**

Colorectal adenocarcinoma (COAD) is a prevalent malignant tumor associated with a high mortality rate. Within the tumor microenvironment, CD8^+^ T cells play a pivotal role in the anti-tumor immune response within the human body. Fibrosis directly and indirectly affects the therapeutic response of tumor immunotherapy. However, the significance of regulatory genes associated with tumor-associated fibrosis and CD8^+^ T cell infiltration remains uncertain. Therefore, it is imperative to identify biomarkers with prognostic value and elucidate the precise role of CD8^+^ T cells and tumor-associated fibrosis.

**Methods:**

We performed a single-cell transcriptome analysis of COAD samples from the GEO database. To evaluate immune infiltration in COAD samples, we utilized CIBERSORT and ESTIMATE. Furthermore, we analyzed the correlation between CD8^+^ T cells and immune infiltration. To analyze COAD expression’s quantitative immune cell composition data, we conducted a Weighted Gene Correlation Network Analysis and utilized a deconvolution algorithm. The data for these analyses were obtained from the GEO database. We utilized univariate Cox regression and LASSO analysis to create a prognostic model. The predictive model was assessed through Kaplan-Meier analysis, and a survival prediction nomogram was created. Additionally, we analyzed the correlation between the prognostic model and chemotherapy drug sensitivity. To estimate the expression of hub genes, we employed immunohistochemistry, real-time PCR, and western blot techniques.

**Results:**

Single-cell transcriptome analysis has indicated a higher prevalence of CD8^+^ T cells in COAD tumor samples. The connection between COAD and CD8^+^ T cells was further confirmed by WGCNA and deconvolution analysis using the GEO database. The Protein-Protein Interaction network analysis revealed three hub genes: *LARS2*, *SEZ6L2*, and *SOX7*. A predictive model was subsequently created using LASSO and univariate COX regression, which included these three genes. Two of these hub genes (*LARS2* and *SEZ6L2*) were found to be upregulated in COAD cell lines and tissues, while *SOX7* was observed to be downregulated. The prognostic model demonstrated a significant association with CD8^+^ T cells, suggesting that these genes could serve as potential biomarkers and targets for gene therapy in treating COAD.

**Conclusion:**

This study has identified three key genes associated with CD8^+^ T cells and the prognosis of COAD, providing new prognostic biomarkers for diagnosing and treating COAD.

## 1 Introduction

Colorectal adenocarcinoma (COAD) has a significant global mortality rate (1). Surgery, chemotherapy, radiotherapy, targeted therapy, and tumor immunotherapy are the leading choices for treating COAD (2). It is worth mentioning that immunotherapy, particularly immune checkpoint inhibitors (ICIs), has shown impressive efficacy in the treatment of non-small cell lung cancer, metastatic melanoma, and prostate cancer (3).

The use of ICIs in the clinical treatment of COAD continues to be greatly limited (4). This limitation is evident from the findings of the Keynote-177 trial, indicating that PD-1 blockade therapy is solely effective for a specific subset of COAD patients, particularly those exhibiting high microsatellite instability or mismatch repair deficiency (5). However, it is essential to mention that around 85% of patients with COAD who have microsatellite stability (MSS) or proficient mismatch repair (PMMR) do not show any response to ICIs) (6).

A crucial molecular mechanism of ICIS resistance involves inadequate infiltration of CD8^+^ T cells or a loss of functionality. CD8^+^ T cells are critical for antitumor immunity, directly mediating tumor cell killing. However, tumors often evade immune responses by suppressing CD8^+^ T cell activity through upregulation of inhibitory molecules such as PD-1 and PD-L1, promoting immune escape and tumor progression (7). Specific cells that suppress the immune system effectively hinder the stimulation of CD8^+^ T cells by adjusting the quantities of PD1 and PDL1, consequently hastening the advancement and decline of COAD. On the other hand, PD-1 inhibits the stimulation of CD8^+^ T cells to enhance T cell defense, resulting in the decline of tumors. Studies have also indicated that the distribution and activity of CD8^+^ T cells significantly influence tumor prognosis and the response to immunotherapy (8). In COAD, a minimal degree of CD8^+^ T cell infiltration at the tumor hub somewhat restricts the efficacy of immunotherapy. Moreover, several studies have confirmed that genes associated with the immune system have a crucial function in the early identification and assessment of prognosis for COAD (9). Therefore, stimulating CD8^+^ T cells may play a crucial role in improving the effectiveness of COAD immunotherapy. Hence, thoroughly investigating biomarkers and related mechanisms of CD8^+^ T cells infiltration and functional irregularities in COAD is of great practical importance to enhance COAD immunotherapy approaches.

Beyond immune cell composition, the tumor microenvironment is heavily influenced by tumor-associated fibrosis, which is increasingly recognized as a major barrier to effective immunotherapy. Tumor-associated fibrosis involves complex and dynamic alterations in the stroma of the tumor microenvironment. Both cellular and non-cellular components of tumor-associated fibrosis collectively or individually impede the efficacy of immunotherapeutic agents. Fibrosis induces the proliferation of connective tissue within tumors, forming a physical barrier that obstructs drug penetration (10). Furthermore, excessive deposition of extracellular matrix (ECM) proteins fosters an immunosuppressive microenvironment (11). Moreover, fibrosis-related factors, such as TGF-β signaling, have been shown to impair T cell function and contribute to tumor progression (12). Despite accumulating evidence highlighting the role of fibrosis in immune evasion, the precise interactions between fibrosis and CD8^+^ T cells in COAD remain poorly understood.

Several studies have explored the molecular mechanisms regulating CD8^+^ T cell infiltration and fibrosis in various cancers, yet a comprehensive analysis integrating single-cell RNA sequencing (scRNA-seq) and gene co-expression network approaches in COAD remains scarce. Existing research primarily focuses on isolated aspects of immune infiltration or fibrosis rather than their combined impact on COAD progression and therapeutic resistance. This study aims to bridge this gap by identifying key molecular biomarkers associated with both CD8^+^ T cell infiltration and tumor fibrosis in COAD.

We initially conducted single-cell transcriptome analysis of COAD samples derived from the GEO database to identify immune-related biomarkers in COAD. This analysis revealed a higher prevalence of CD8^+^ T cells in COAD samples than in healthy samples. Afterward, we performed a WGCNA on COAD gene-level data. In addition, we employed the CIBERSORT algorithm for relative subset estimation to analyze RNA transcripts. This was done to understand the composition of immune cells in COAD samples. We used each patient’s immune cell content as a characteristic input, which was then combined with mRNA expression data to construct a WGCNA network. This network enabled the identification of module genes with the highest correlation to immune infiltration, and further exploration of their molecular mechanisms was undertaken (13). Finally, we validated the biomarkers associated with immune prognosis.

## 2 Materials and method

### 2.1 Single-cell RNA sequencing data collection and preprocessing

ScRNA-seq of COAD patients in Lee et al. study were obtained from GEO database (GSE132465) (14). GSE132465 is a single-cell RNA sequencing dataset related to COAD, providing data on 63689 cells from 23 COAD patients. These cell samples include 23 primary COAD samples and 10 matched normal mucosal tissue samples. R packages ‘Seurat’ was used, followed by ‘LogNormalize’ cell normalization and scale (15). PCA was performed on the top 1,000 genes with high variability. Then, the top 40 principal components were chosen for clustering with a resolution of 0.3. Cells were visualized using the t-SNE method, which employs a two-dimensional t-distributed stochastic neighbor embedding and the same distance metric. The function ‘FindAllMarkers’ was utilized to screen marker genes using a logfc. Threshold of 0.25, a min.pct of 0.1, and a p_val_adj of 0.05. The Human Primary Cell Atlas Data provided by the ‘celldex’ package was used to annotate cell types using the ‘SingleR’ package (16).

### 2.2 Identification of T cell subpopulations

We extracted the T cells labeled by SingleR and identified their subgroups. The classic workflow of Seurat was employed to perform dimensionality reduction and unsupervised clustering analysis on the top 15 principal components. Within each cluster, we selected the top 10 differentially expressed genes (DEGs) as markers. Cell types were manually annotated based on the cell markers recorded in the CellMarker 2.0 database (http://117.50.127.228/CellMarker/). The criteria for cell type annotation was as follows: firstly, we annotated the clusters using markers specific to the “Colon” and “Colorectum” tissues in CellMarker 2.0; secondly, when accurate labels were lacking, we considered markers labeled as “Immune system” in a comprehensive manner; thirdly, for other cases, we took into account various markers for cell type annotation (17). Then, the immune markers in other solid tissue, which histological location are close to the colorectal, were used for annotation.

### 2.3 Analysis of intercellular communication

The cellular communications among cell types were analyzed using the R package ‘CellChat.’ Our primary attention was directed towards the human database in CellChat, where we utilized the functions ‘identifyOverExpressedGenes’ and ‘identifyOverExpressedInteractions’ to detect ligands or receptors expressed at higher levels. Then, we mapped expression data onto the Protein-protein interaction network. The functions ‘computeCommunProb’ and ‘filterCommunication’ were employed to calculate the probability of communication and deduce the cellular communication network. The functions ‘computeCommunProbPathway’ and ‘aggregateNet’ were employed to deduce the intercellular communication among every type of cell (18).

### 2.4 Weighted gene co-expression network analysis

We downloaded GSE17536, GSE29621, and GSE74604 datasets from NCBI GEO with GPL570 and GPL6104 annotations, respectively. These datasets contain transcriptome data of COAD patients. We then combined and corrected data using the ‘SVA’ algorithm. Finally, we downloaded COAD mRNA expression data, extracting 433 samples with complete profiles and survival time > 0.

We aim to analyze the relationship between gene networks and phenotypes by constructing a network of co-expressed genes and identifying modules with different weights. We utilize the WGCNA R package to structure the co-expression network (19). Applying the algorithm to the top 5000 genes exhibiting the greatest variance, a soft threshold of 4 is set. After that, the weighted adjacency matrix is converted into a topological overlap matrix (TOM) to measure network connectivity. The various branches of the clustering tree represent different gene modules, which are indicated by different colors to represent separate modules. Modules are formed by grouping genes based on their expression patterns, which are determined by calculating the weighted correlation coefficient of genes. Consequently, multiple modules are formed from tens of thousands of genes based on their respective gene expression patterns. (20).

### 2.5 Analysis of immune cell infiltration

The RNA-seq data of COAD patients is subjected to CIBERSORT analysis to determine the proportions of 22 immune infiltrating cells. CIBERSORT is an algorithm based on gene expression profiles that estimates the relative proportions of 22 immune cell types. To run CIBERSORT, we need to download the “LM22.txt” file from the website (https://cibersort.stanford.edu/download.php). The LM22.txt file is a “signature matrix” containing 547 genes and 22 immune cell types. Additionally, immune and stromal scores for each sample were calculated using the “estimate” R package. The immune cells of each patient are input as features. Then, modules of genes closely related to immune infiltration are identified using the WGCNA network and mRNA expression data. Further research is conducted on their molecular mechanisms (21).

### 2.6 Analyzing the enrichment of functions in gene modules

The Metascape database (www.metascape.org) was used to annotate and visualize module genes, revealing the biological functions and signaling pathways linked to the initiation and advancement of diseases. Specific genes underwent analysis using Gene Ontology (GO) and Kyoto Encyclopedia of Genes and Genomes (KEGG) pathway analysis. To establish statistical significance, a minimum overlap of 3 and a p-value less than or equal to 0.01 were considered (22).

### 2.7 The relationship between hub genes and immune cells

The TIMER database is a powerful resource for examining RNA-Seq expression spectrum data, particularly for immune cells. Using the TIMER database, we explored the relationship between the central gene and the population of immune cells. Additionally, we examined the variation in tumor infiltration (23).

### 2.8 Drug sensitivity analysis

Using the Cancer Drug Sensitivity Genomics Database (GDSC, https://www.cancerrxgene.org/), we employed the R package ‘pRRophetic’ to forecast the sensitivity of the tumor sample to chemotherapy. Regression analysis obtained the IC50 estimates for each chemotherapy drug treatment. The regression and prediction accuracy were assessed via a 10-fold cross-validation utilizing the GDSC training set. All parameters were maintained at their regular settings, which included the application of “combat” to eliminate batch effects and the averaging of expression values of duplicate genes.

### 2.9 Gene Set Enrichment Analysis

The Gene Set Enrichment Analysis (GSEA) is a research technique that categorizes genes by contrasting the variances in gene expression, utilizing pre-established gene sets derived from the KEGG database. Afterward, it assesses if the predetermined gene sets exhibit enrichment in either the upper or lower portion of the ranking. This study employed the GSEA technique to examine the central genes of signaling pathways in cohorts exhibiting significant variations in gene expression. The aim was to investigate the underlying molecular mechanisms responsible for prognostic disparities between the two groups and ascertain the involvement of hub genes in tumor advancement.

### 2.10 Cell culture

The Institute of Cell Biology supplied human colon carcinoma cell lines Lovo, HCT116, HT29, SW480, and SW620, and a healthy human colon cell line, NCM460. NCM460, HCT116, HT29, and SW620 cell lines were grown in RPMI-1640 medium supplemented with 10% fetal calf serum, 100 U/ml of penicillin, and 100 μg/ml of streptomycin. The Lovo cell line was cultured in an F-12K medium under the same conditions. The cultures were maintained in a 37°C incubator with a 5% CO2 atmosphere. The SW480 cell line was grown in Leibovitz’s L-15 medium with identical additives but under 100% air conditions at a temperature of 37°C.

### 2.11 Clinical samples

10 COAD and healthy colon tissues were resected at the Second Affiliated Hospital of Harbin Medical University between 2022 and 2023. The gathering and use of these tissues followed the moral guidelines specified in the Declaration of Helsinki. Obtained written consent from every patient, which was approved by the Research Ethics Committee of the Second Affiliated Hospital of Haibin Medical University.

### 2.12 Immunohistochemistry staining

The Immunohistochemistry (IHC) experiment was conducted using a two-step method. To begin, the tissue sections embedded in paraffin were dewaxed and subsequently rehydrated utilizing a gradient of alcohol. Following this, they were subjected to boiling for 15 minutes in a citrate buffer with a pH of 6.0. Peroxidase activity was inhibited for 20 minutes to avoid non-specific staining. The sections were rinsed three times with PBS, then incubated overnight with *SOX7* antibody (RD, AF2766, 1:200 dilution), *LARS2* antibody (GeneTex, GTX110083, 1:200 dilution), and *SEZ6L2* antibody (RD, AF4916, 1:200 dilution) at 4°C. Afterward, the sections were rinsed thrice with PBS and then incubated with a reaction enhancer kit at 20°C for about 20 minutes. After being washed in PBS three times, they were incubated with secondary antibodies for approximately 20 minutes at 20°C then stained with 3,3-diaminobenzidine. Finally, the sections were dehydrated, sealed, and restained with hematoxylin.

### 2.13 RNA extraction and qRT-PCR

The procedures were conducted in published articles per the manufacturer’s recommendations. Cells were used to extract total RNA with TRIpure reagent (BioTeke, RP1001). The PrimeScript RT reagent kit (Perfect RealTime) from Takara Biotechnology Ltd was utilized to convert total RNA to cDNA. Beta Actin was chosen as an internal reference. RT-qPCR assays were performed using an Exicycler 96 qPCR instrument (BIONEER Company). The primer set for *LARS2* was F: ACACGATTCGGCTCTAC and R: TCCTCTGTGAAATGGGT.The primer set for *SOX7* was F: ACGCCTTCATGGTTTGGG and R: CTTGGCCTGCTTCTTCCTG.The primer set for *SEZ6L2* was F: TCCGACATTCTCACTTGCC and R: GGACCCTATCGCTCCACTT.The primer set for Beta Actin was F: GGCACCCAGCACAATGAA and R: TAGAAGCATTTGCGGTGG.The 2-ΔΔCt formula was utilized to analyze the comparative level of expression.

### 2.14 Western blot analysis

The procedure for immunoblotting includes the homogenization and ultrasonic treatment of cells in RIPA buffer. Small volumes of supernatant are collected using the Bradford method, and the protein content is determined by measuring the absorbance with a visible spectrophotometer. A standard curve is drawn using BSA as a standard control; then, the extracted proteins are collected for electrophoresis. Following electrophoresis, the samples are moved onto a PVDF membrane and obstructed using skim milk. Following the wash, the membrane underwent overnight oscillation at 4°C using a primary antibody (*SOX7* antibody-RD, AF2766 at a dilution of 1:500; *LARS2* antibody-GeneTex, GTX110083 at a dilution of 1:1000; *SEZ6L2* antibody-RD, AF4916 at a dilution of 1:500; or β-actin antibody-wanleibio, WL01372/WL01114 at a dilution of 1:1000). Next, a secondary antibody (*SOX7* conjugated rabbit anti-goat IgG-Abclonal AS029, at a dilution of 1:5000; *LARS2* conjugated goat anti-rabbit IgG-wanleibio WLA023, at a dilution of 1:5000; *SEZ6L2* conjugated rabbit anti-sheep IgG-Abclonal AS023, 1:5000 dilution; β-actin conjugated goat anti-rabbit IgG-wanleibio WLA023, at a dilution of 1:5000) was introduced and left to incubate at room temperature for 2 hours. Following three washes of the cell membrane, the immunoblot analysis was conducted using chemiluminescence. Finally, the membrane was incubated in ECL-HRP reagent for 1 minute and then developed in a visualizer.

### 2.15 Statistical analysis

GraphPad Prism 8 and R 3.6.3 (https://www.r-project.org/) were utilized for the statistical analysis. In the comparison of continuous variables in the immunohistochemical analysis, the Student’s t-test was used. In our study, we utilized several statistical methods to construct and validate the prognostic model. Univariate Cox regression analysis was performed using the survival package in R to assess the association between gene expression and overall survival (OS) in COAD patients. Hazard ratios (HRs) and 95% confidence intervals (CIs) were calculated, and statistical significance was determined using the log-rank test, with genes meeting the threshold of p < 0.05 being considered for further analysis. To refine the selection of prognostic genes and prevent overfitting, we applied LASSO regression using the glmnet package in R. The LASSO approach, which incorporates 10-fold cross-validation, enabled the identification of the most predictive genes while removing redundant ones, optimizing the prognostic model. Kaplan-Meier survival analysis was then conducted to evaluate the survival differences between patient subgroups stratified by gene expression levels, with survival curves generated using the survfit function in the survival package. The log-rank test was applied to compare survival distributions, with a p-value < 0.05 indicating statistically significant differences. Furthermore, to validate the predictive performance of our model, we constructed a nomogram and assessed its accuracy using receiver operating characteristic (ROC) curve analysis, where the area under the curve (AUC) was calculated to evaluate the prognostic efficacy of the gene signature. These statistical approaches ensured that our model was both robust and clinically relevant.

## 3 Results

### 3.1 Resolution of the immune microenvironment in COAD

**Figure 1:**
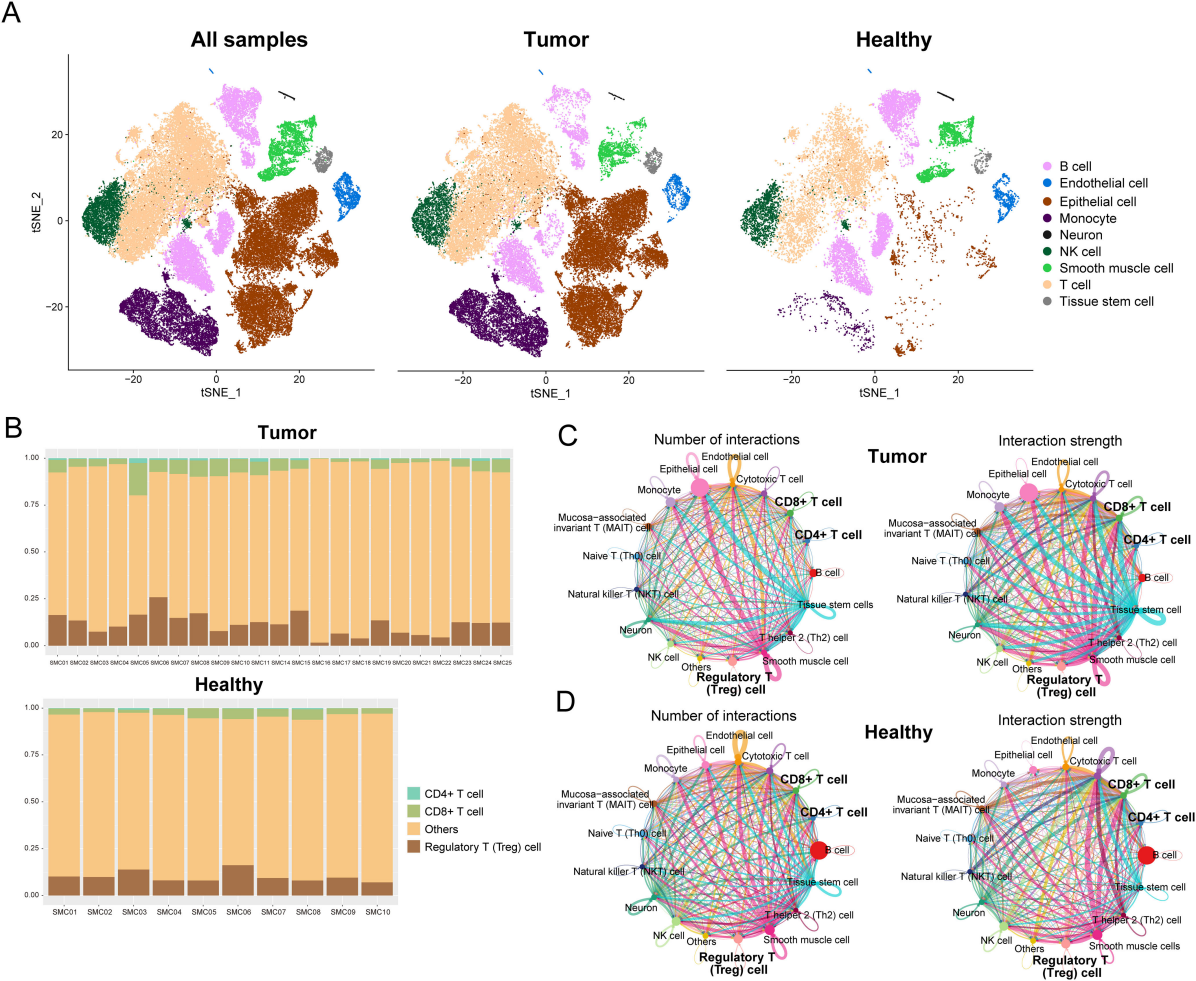
Resolution of the immune microenvironment in COAD and matched normal samples by scRNA-seq. **(A)** Cells were clustered into nine types via the tSNE dimensionality reduction algorithm; each color represented the annotated phenotype of each cluster. **(B)** Proportions of CD8^+^ T cells, CD4^+^ T cells, and Treg cells in each sample or tumor type. **(C, D)** Numbers and strength of interactions among cell types in COAD and healthy single cells.

We acquired scRNA-seq data from GEO for 23 COAD samples and 10 corresponding healthy samples. The Seurat workflow (**Supplementary Figure 1A**) was used to preprocess the gene expression matrix. A strong positive correlation (Pearson’s correlation = 0.9, **Supplementary Figure 1B**) was observed between the number of detected genes (nFeature) and sequencing depth (total number of UMIs, nCount). We identified DEGs in each cluster by the FindAllMarkers’function, and the top five significant DEGs of clusters were visualized using a heat map (**Supplementary Figure 1C**). Using the SingleR method, the cell types in COAD and healthy samples were annotated (**Figure 1A**). The primary cell types consisted of B cells, endothelial cells, epithelial cells, monocytes, neurons, NK cells, smooth muscle cells, T cells, and tissue stem cells. We further annotated T cell subtypes and observed differences in their proportions between tumor and healthy samples (**Figure 1B**). Quantitative analysis revealed that tumor samples contained significantly higher percentages of CD8^+^ T cells (tumor: 15.3% vs. healthy: 9.1%, p=0.003), CD4^+^ T cells (tumor: 18.7% vs. healthy: 12.4%, p=0.005), and regulatory T (Treg) cells (tumor: 7.8% vs. healthy: 2.1%, p<0.001) compared to healthy samples. The most pronounced difference was observed in Treg cells, which showed a 3.7-fold increase in tumor sample.

Next, we performed a cell-cell communication analysis using CellChat to explore the mutual effects in the tumor microenvironment. The numbers and strength of interactions among cell types in tumor and healthy samples are shown in **Figures 1C, D**. The number of cellular communications received by epithelial cells in tumor samples were more than in healthy samples. Compared to healthy samples, both the number and strength of cellular communications received by CD8^+^ T cells and cytotoxic T cells were reduced (**Supplementary Figure 2**). Infiltration of CD8^+^ T cells is associated with patients’ prognosis in colorectal cancer.

### 3.2 Gene co-expression network analysis reveals distinct modules associated with T cell infiltration in COAD

**Figure 2:**
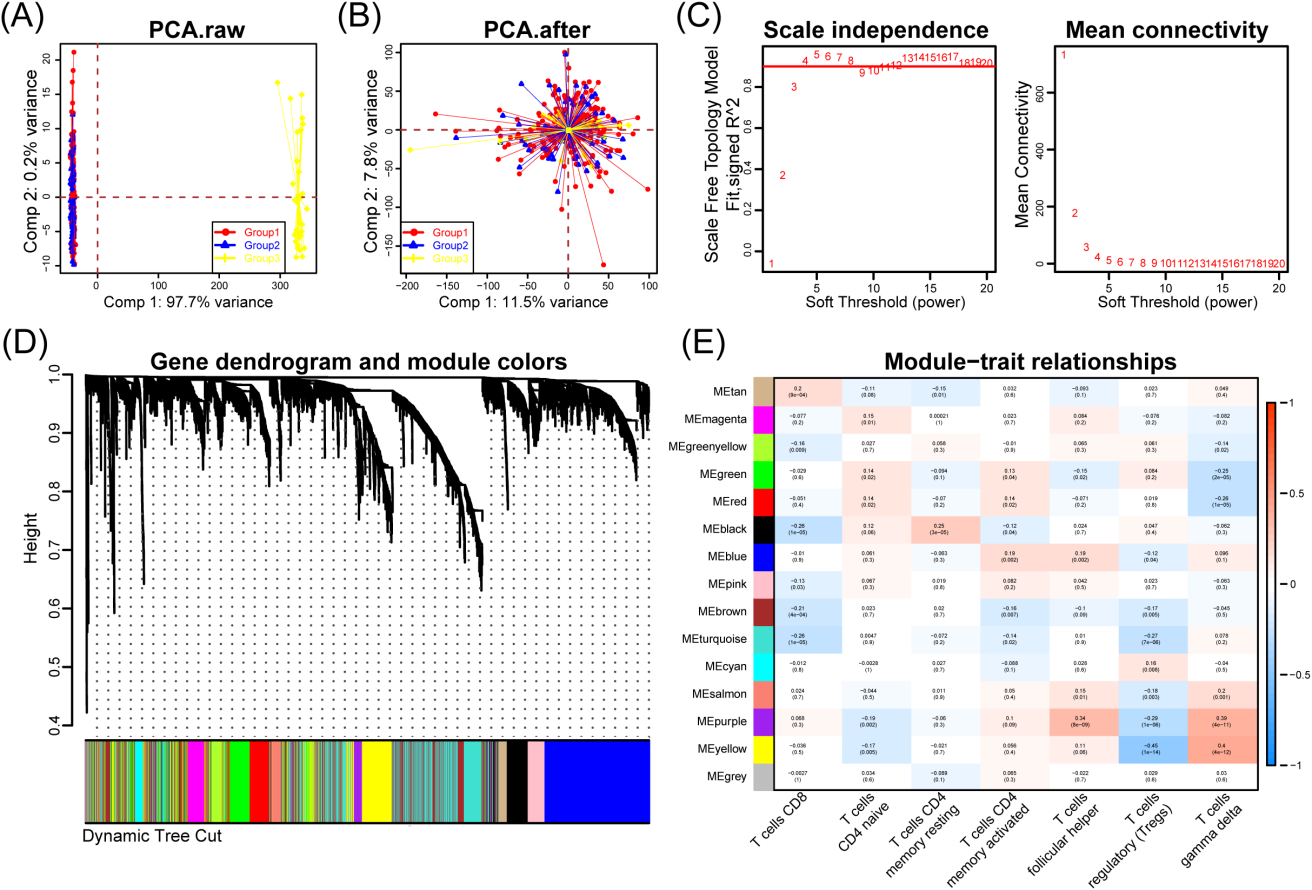
Weighted gene co-expression network analysis (WGCNA) for hub module construction and validation. **(A, B)** PCA plots showing batch effects before and after correction using the SVA algorithm. **(C)** Soft-threshold power selection for scale-free network construction, depicting the scale-free topology model fit and mean connectivity. **(D)** Hierarchical clustering dendrogram of genes based on topological overlap, with distinct color-coded modules. **(E)** Module-trait relationships, with heatmaps demonstrating strong correlation between black/turquoise modules and CD8^+^ T cell infiltration (p < 0.05).

Analysis of 272 COAD samples from the GEO database uncovered significant patterns of gene co-expression associated with immune cell infiltration. After applying the SVA algorithm to eliminate batch effects (**Figures 2A, B**), we constructed a gene co-expression network using WGCNA. This network analysis revealed 15 distinct gene modules with unique expression patterns and biological functions (**Figure 2D**).

Notably, we identified a strong negative correlation between the black module and CD8^+^ T cell infiltration (correlation coefficient = -0.26, p-value = 1e-05), suggesting that genes within this module may play crucial roles in regulating CD8^+^ T cell recruitment or function within the tumor microenvironment (**Figure 2E**). This inverse relationship indicates that as expression of black module genes increases, CD8^+^ T cell infiltration decreases, potentially identifying a mechanism of immune evasion in colorectal adenocarcinoma. The turquoise module also demonstrated significant association with CD8^+^ T cells, highlighting multiple gene networks involved in modulating tumor immunity in COAD.

### 3.3 Hub modules reveal key biological processes mediating T cell-tumor interactions

**Figure 3:**
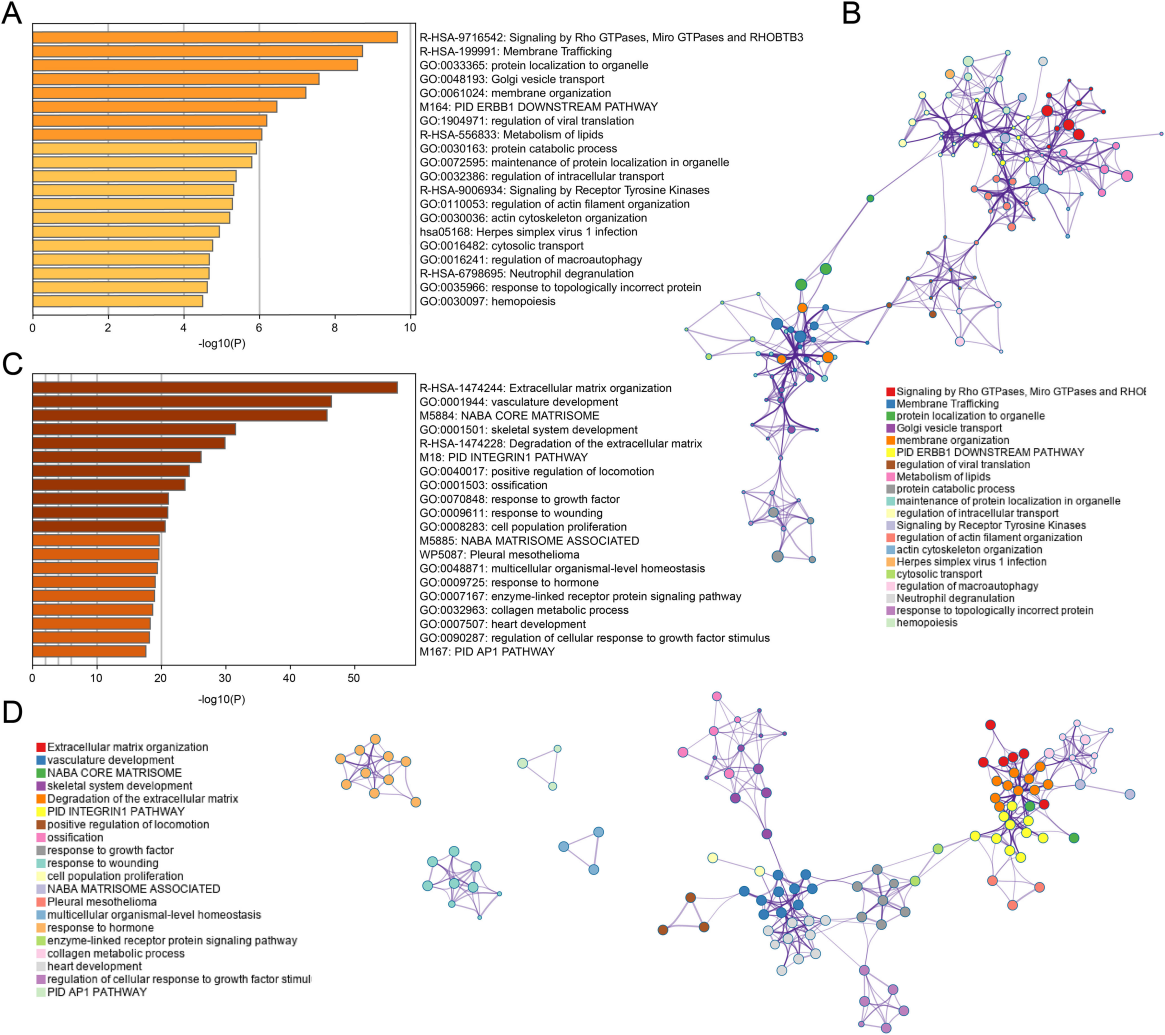
Enrichment analysis for black and turquoise modules. **(A, B)** GO and KEGG enrichment analysis for black module genes, revealing significant pathways in membrane trafficking, protein localization, and metabolism. **(C, D)** Enrichment analysis for turquoise module genes, highlighting pathways involved in ECM remodeling, vascular development, and growth factor signaling.

Functional enrichment analysis of the black module revealed significant enrichment in pathways related to Rho GTPase signaling, membrane trafficking, and lipid metabolism (**Figure 3A**). This suggests that tumor cells may modulate their cytoskeletal dynamics, vesicular transport, and metabolic profiles to create an immunosuppressive microenvironment. The involvement of Receptor Tyrosine Kinase signaling further points to potential therapeutic targets for enhancing T cell infiltration, as these kinases are known regulators of immune checkpoint expression and function.

In contrast, the turquoise module genes were primarily associated with extracellular matrix organization, vasculature development, and growth factor responses (**Figure 3C**). This indicates that physical barriers created by matrix remodeling and aberrant angiogenesis likely contribute to CD8^+^ T cell exclusion from tumor sites. The enrichment in hormone response pathways also suggests a potential link between endocrine factors and immune regulation in COAD, which has not been extensively explored previously.

The network visualization (**Figures 3B, D**) demonstrates how these functional pathways interconnect, revealing potential hub genes that coordinate multiple aspects of tumor-immune interactions. These molecular signatures may serve as prognostic indicators and identify novel therapeutic targets to enhance anti-tumor immunity in colorectal cancer patients.

### 3.4 Identification and verification of hub genes

**Figure 4:**
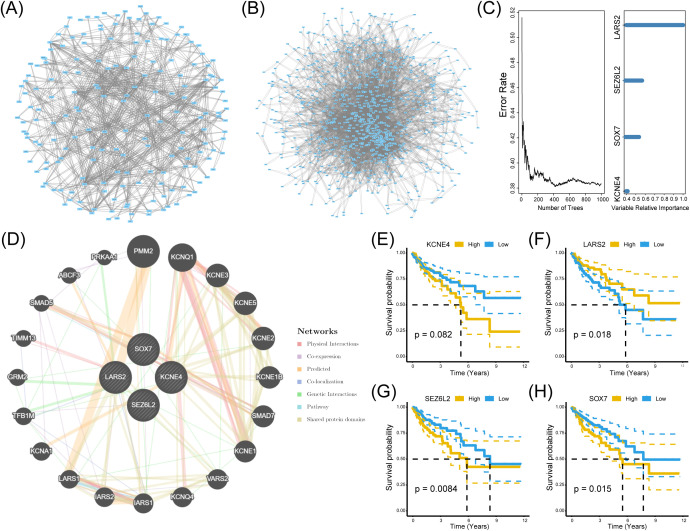
Identification of significant genes related to prognosis. **(A, B)** PPI networks of genes in the black and turquoise modules. **(C)** Random survival forest analysis using TCGA data, identifying genes with a relative importance > 0.4 as prognostic markers. **(D)** GeneMANIA interaction network illustrating functional relationships among the four hub genes. **(E-H)** Kaplan-Meier survival curves for *LARS2, SEZ6L2*, and *SOX7*, demonstrating significant associations between high/low expression levels and patient survival outcomes (log-rank test, p < 0.05).

To investigate the hub genes associated with CD8^+^ T cells infiltration, we built a protein-protein interaction (PPI) network using the String data frame (with a minimum interaction value of 0.4). We visualized it through Cytoscape software (**Figures 4A, B**). Afterward, we utilized the TCGA dataset to examine the random survival forest of genes in two modules. We regarded genes with relative importance exceeding 0.4 as the ultimate markers and assessed the significance of the four genes (**Figure 4C**).

Furthermore, GeneMANIA demonstrated a substantial interaction among the four genes (**Figure 4D**). Consequently, these four genes were designated HUB genes in both analyses to elucidate their relationship with CD8^+^ T cells. In pursuit of identifying biomarkers influencing the prognosis of COAD, we analyzed the survival of these four HUB genes utilizing the TCGA dataset. The Kaplan-Meier plotter database assessed the prognostic significance of *KCNE4*, *SOX7*, *SEZ6L2*, and *LARS2*. Our findings revealed a significant negative correlation between *SEZ6L2* and *SOX7* with the prognosis of COAD, while *LARS2* demonstrated a positive correlation. The results for *KCNE4* (p=0.082) were not statistically significant. In summary, we propose *LARS2* (p=0.018), *SEZ6L2* (p=0.0084), and *SOX7* (p=0.015) as prognostic biomarkers for COAD (**Figures 4E–H**).

### 3.5 Determination of immunological and clinical characteristics

**Figure 5:**
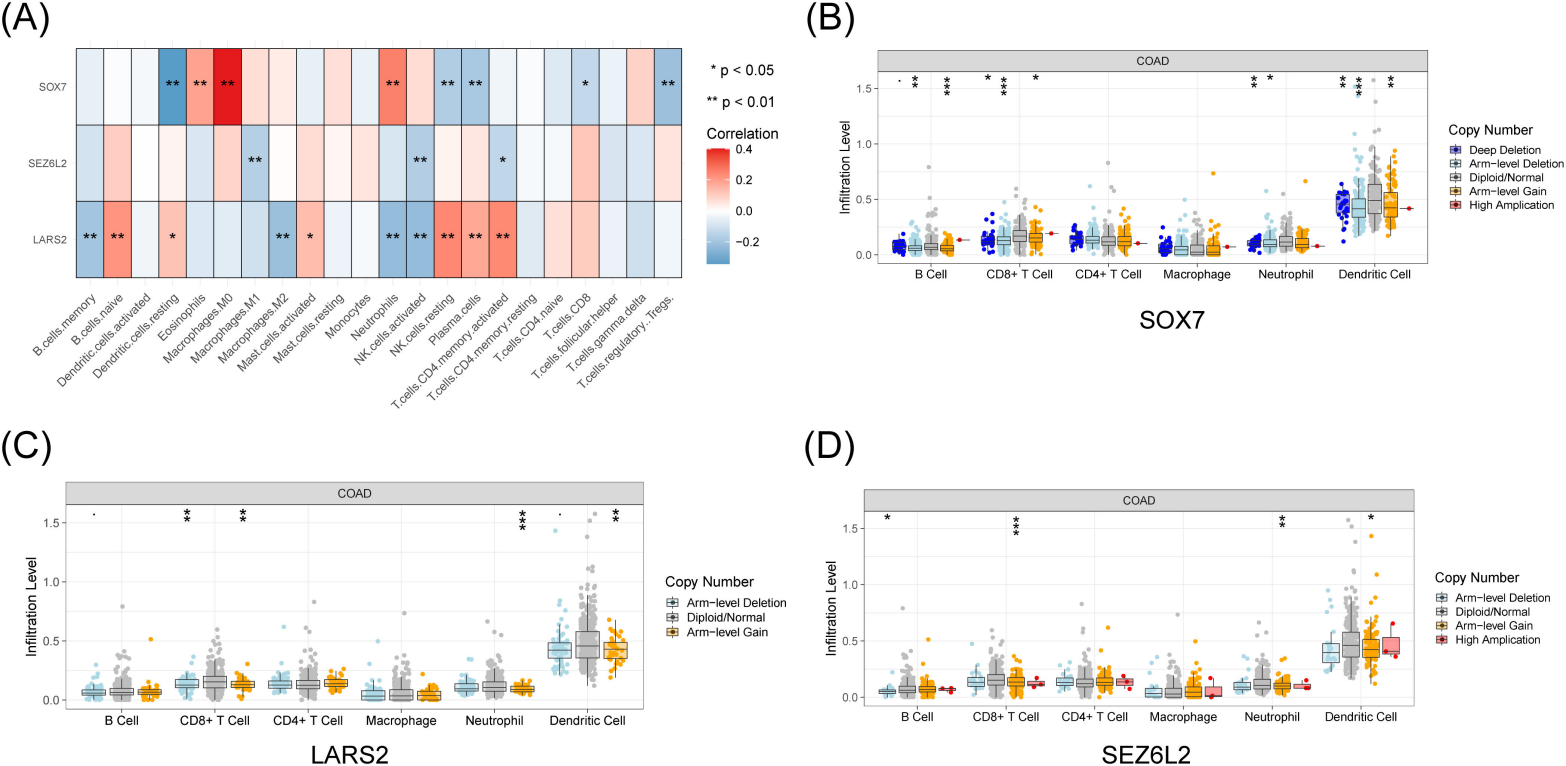
Immune and clinical characteristics of these hub genes. **(A)** Expression profiles of *LARS2, SEZ6L2*, and *SOX7* across different immune cell populations from TCGA data. **(B-D)** Immune infiltration analysis from the TIMER database, showing the correlation between hub gene expression levels and CD8^+^ T cell infiltration (p < 0.05). * represents a *p*-value < 0.05, ** represents a *p*-value < 0.01, and *** represents a *p*-value < 0.001. The smaller the *p*-value, the more statistically significant the result.

We thoroughly investigated these pivotal artificial intelligence genes in light of the substantial immunological and clinical attributes of the three central genes, *LARS2*, *SEZ6L2*, and *SOX7*. By utilizing the TIMER database, we obtained the expression levels of these critical genes, unveiling different numbers of copies in diverse immune cell types (**Figure 5A**). Moreover, we discerned a correlation between the expression profiles of these three artificial intelligence genes and the infiltration level of CD8^+^ T cells (**Figures 5B–D**). The results indicate that these three core genes closely connect with the infiltration of CD8^+^ T cells and play a role in creating the immune microenvironment. The analysis of *LARS2*, *SEZ6L2*, and *SOX7* in the COAD tumor microenvironment suggests that these genes play significant roles in regulating CD8^+^ T cell infiltration and function. While *LARS2* and *SEZ6L2* may contribute to immune suppression and tumor immune evasion, *SOX7* appears to promote CD8^+^ T cell infiltration and antitumor immunity.

### 3.6 Hub gene set enrichment analysis

**Figure 6:**
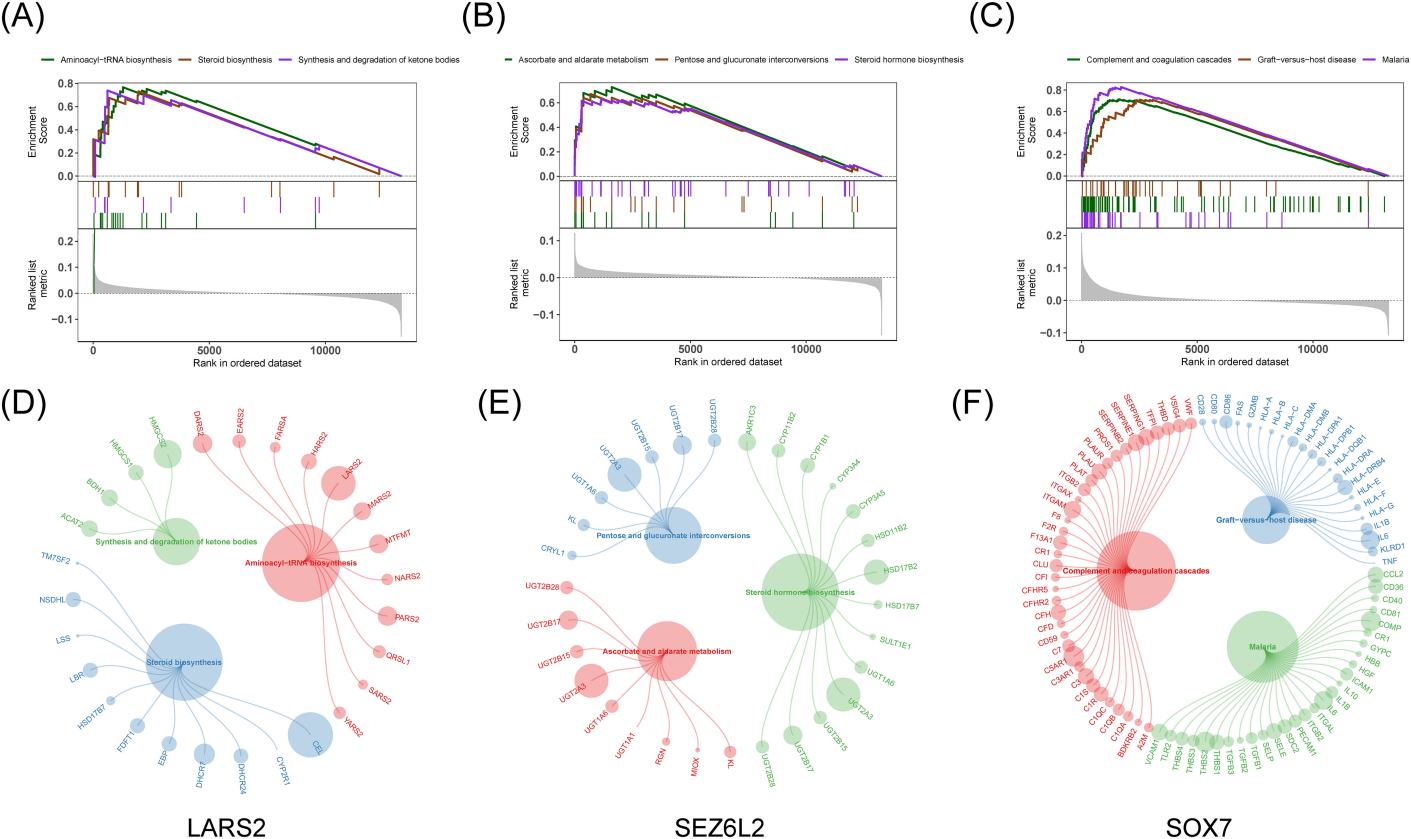
Gene Set Enrichment Analysis for hub genes. **(A-C)** GSEA enrichment plots for top three signaling pathways related to *LARS2, SEZ6L2*, and *SOX7*. **(D-F)** Bubble plots highlighting key genes involved in the enriched pathways, where larger circles represent higher enrichment scores.

Afterward, we examined the particular signaling pathways implicated in three predictive biomarkers and investigated their possible molecular mechanisms influencing the advancement of COAD. Among them, the pathways significantly enriched in *LARS2* are aminoacyl-tRNA biosynthesis, steroid biosynthesis, and ketone synthesis and degradation. The pathways for *SEZ6L2* are ascorbic acid and malate metabolism, pentose and glucuronate interconversions, and steroid hormone biosynthesis. As for *SOX7*, significantly enriched pathways are complementary and coagulation cascades, graft-versus-host reaction, and malaria (**Figures 6A–F**).

Besides, we obtained drug-gene and PPI associated with hub genes from the Pathway Commons database (https://www.pathwaycommons.org/) and constructed an interacted network. *SEZ6L2* and *SOX7* shared plentiful drugs, *LARS2* and *SOX7* shared three drugs, and *LARS2* and *SEZ6L2* shared common neighbor genes (e.g., *NTRK1*) and drugs, suggesting the functional correlation among hub genes (**Supplementary Figure 3**).

### 3.7 Identification of prognostic biomarkers

**Figure 7:**
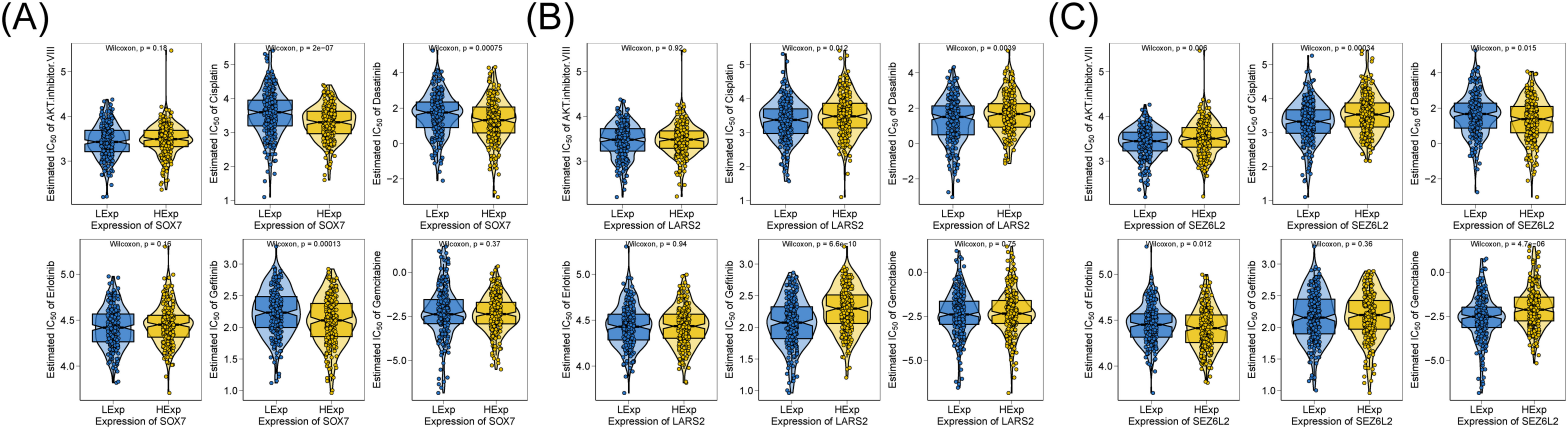
Drug sensitivity data is based on the GDSC database. **(A-C)** Correlation of hub gene expression with drug response in the GDSC database. Results indicate that *SEZ6L2* and *SOX7* are associated with sensitivity to cisplatin, dasatinib, and gefitinib, while *LARS2* expression correlates with response to cisplatin and gemcitabine.

A thorough examination was conducted to identify biomarkers that impact the prognosis of COAD, considering the significant immune and clinical features of the three central genes. The efficacy of COAD surgery in combination with chemotherapy is well established. The analysis depends on drug sensitivity information obtained from the GDSC database, using the R software package ‘pRRophetic’ to forecast the responsiveness to chemotherapy for every tumor specimen. This enables further exploration of the sensitivity of the hub genes and commonly used chemotherapeutic agents. The findings reveal a significant correlation between *LARS2* and *SEZ6L2* concerning the sensitivity to Cisplatin, Dasatinib, and Gemcitabine, while *SOX7* demonstrates a similar correlation with the sensitivity to Cisplatin, Dasatinib, and Gefitinib (**Figures 7A–C**).

**Figure 8:**
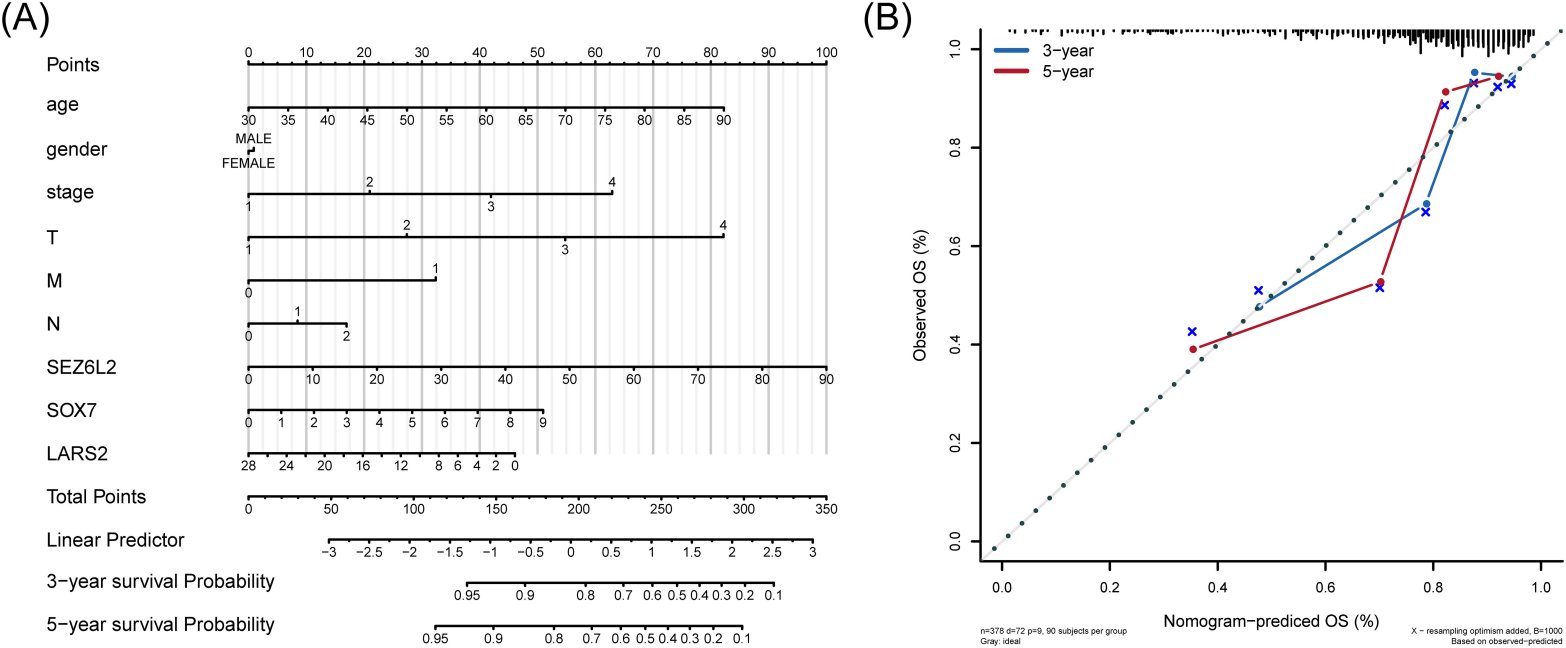
Nomogram for predicting overall survival in COAD patients. **(A)** Nomogram-based prognostic scoring system, integrating clinical factors and hub gene expression for 3-year and 5-year survival prediction. **(B)** Calibration curves demonstrating the high predictive accuracy of the nomogram (AUC = 0.72), compared to TNM staging (AUC range: 0.62–0.68).

A line graph presented the regression analysis results performed on COAD samples. The logistic regression analysis results show that the distribution of different clinical indicators of COAD in our samples has variably influenced the overall scoring process (**Figure 8A**). At the same time, we also predicted and analyzed the survival status within three and five years. The results show that the predictive efficiency of the nomogram model is higher (**Figure 8B**).

To calculate the discriminate performance of the nomogram and the TNM stage system, we used a ROC to compare the prognostic capabilities in the TCGA dataset (**Supplementary Figure 4**). The area under the nomogram’s AUC is 0.72, and 0.62, 0.66, 0.63, 0.68 of T stage, N stage, M stage, and Stage respectively.

### 3.8 Experimental validation of hub genes

**Figure 9:**
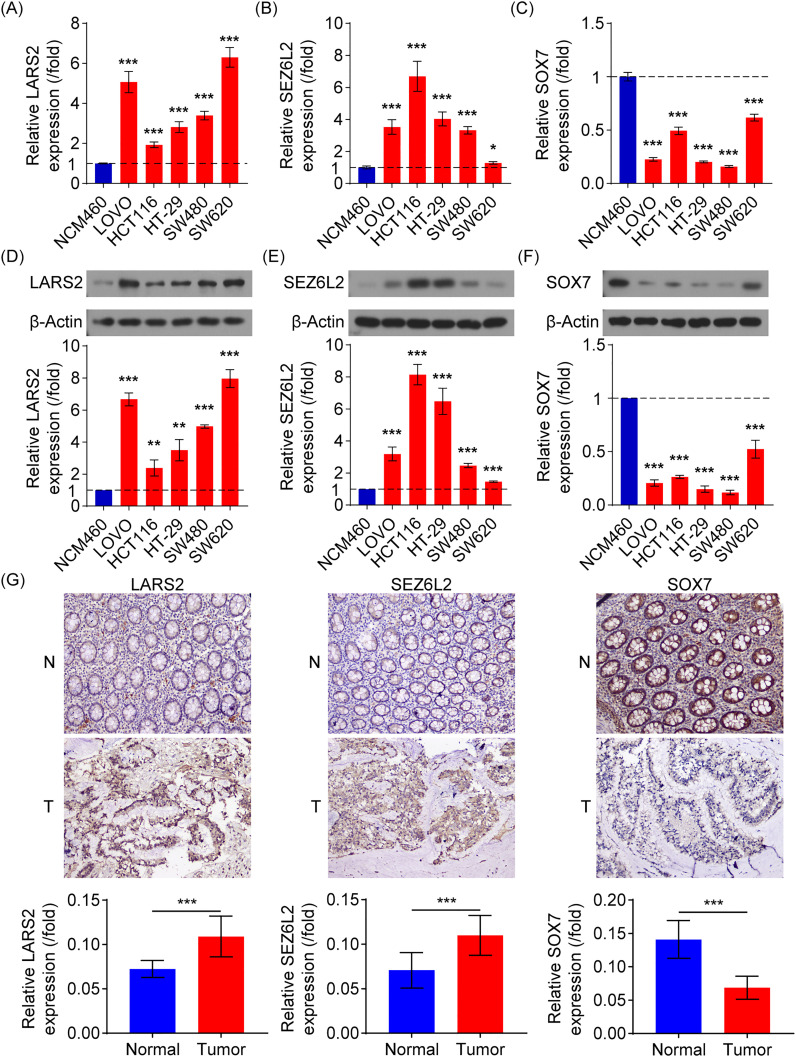
Experimental verification of signature gene expression in COAD. **(A-C)** RT-qPCR analysis of *LARS2, SEZ6L2*, and *SOX7* mRNA expression in COAD and normal colonic epithelial cell lines. **(D-F)** Western blot analysis confirming higher *LARS2* and *SEZ6L2* protein levels and lower SOX7 protein expression in COAD cells compared to normal cells. **(G)** IHC analysis of *LARS2*, *SEZ6L2*, and *SOX7* in COAD patient tissues, showing significant overexpression of *LARS2 and SEZ6L2*, and downregulation of *SOX7* compared to matched normal tissues (p < 0.001). * represents a *p*-value < 0.05, ** represents a *p*-value < 0.01, and *** represents a *p*-value < 0.001. The smaller the *p*-value, the more statistically significant the result.

Various molecular experiments were conducted to assess the clinical relevance of the central genes *LARS2*, *SEZ6L2*, and *SOX7*. The focus was placed on evaluating both mRNA expression and protein levels of these genes in the NCM460 healthy colonic epithelial cell line and five COAD cell lines (Lovo, HCT116, HT29, SW480, and SW620). The findings demonstrated that *LARS2* and *SEZ6L2* exhibited significantly higher expression levels in COAD cell lines, whereas *SOX7* expression was markedly reduced (**Figures 9A–C**). These results indicate that *LARS2* and *SEZ6L2* may play oncogenic roles in COAD, while *SOX7* may function as a tumor suppressor. To validate these findings at the protein level, Western blot analysis was performed. The results were consistent with the gene expression data, confirming the upregulation of *LARS2* and *SEZ6L2* and the downregulation of *SOX7* in COAD cells (**Figures 9D–F**). These molecular alterations suggest that *LARS2* and *SEZ6L2* might contribute to COAD progression by promoting tumor growth and immune evasion, whereas *SOX7* downregulation may facilitate tumor development by impairing tumor suppressive pathways.

### 3.9 Immunohistochemical pathological analysis

To further investigate the biological significance of these genes in COAD progression, we performed IHC analysis using tumor and adjacent normal tissues from 10 COAD patients. Image analysis software was used to quantify staining intensity for *LARS2*, *SEZ6L2*, and *SOX7*. Consistent with the *in vitro* findings, the IHC results revealed that *LARS2* and *SEZ6L2* were highly expressed in COAD tissues, while *SOX7* exhibited significantly lower expression levels compared to normal tissues (**Figure 9G**).

## 4 Discussion

The growth and spread of tumors are closely connected to the human immune system, as immune responses can impact their development and metastasis (1). Lymphocytes specific to tumors are pivotal in this process and significantly correlate with anti-tumor treatments. CD8^+^ T cells, possessing tumor antigen specificity and the ability to destroy tumor cells, are extensively utilized in immunotherapy and the creation of tumor vaccines (7). Research has substantiated that CD8^+^ T cells can target and eliminate tumor cells within the microenvironment, thus serving an anti-tumor function. Moreover, the quantity of CD8^+^ T cells is directly proportional to the survival duration of tumor patients (24). T cells within the tumor cell microenvironment express PD-1 receptors that interact with PD-L1 molecules expressed by tumor cells. This activates the PD-1 pathway, inhibiting T cells from destroying tumor cells and facilitating immune evasion by tumor cells. Immunotherapy with PD-1/PD-L1 inhibitors, which obstruct the binding of PD-1 and PD-L1, can amplify the body’s anti-tumor mechanisms and achieve therapeutic effects. (25, 26). This study acquired scRNA-seq data from 23 COAD samples and 10 corresponding healthy samples (GSE132465) from the GEO database. The findings indicated that the infiltration rate of CD8^+^ T cells in COAD samples exceeded that in healthy intestinal tissue, and there was a variation in the degree of CD8^+^ T cell infiltration among different pathological types. Therefore, examining and researching genes linked to the infiltration of CD8^+^ T cells can somewhat predict the prognosis and advancement of the condition in individuals.

Fibrosis is a pathological condition primarily caused by the persistent presence of chronic inflammation, which leads to excessive tissue remodeling and repair. This phenomenon is somewhat analogous to the healthy wound healing process. In areas surrounding tumors, fibroblasts originally present in the tissue are recruited. Under the influence of cytokines such as TGF-β, these cells differentiate into myofibroblast-like cells (27). These activated myofibroblasts produce large amounts of ECM components. However, the excessive production of ECM accumulates during tumor-associated fibrosis, creating interference that impedes the effectiveness of immunotherapy (28). As a non-cellular component, the extracellular matrix provides a physical barrier to the effective penetration of drugs. The presence of this barrier makes it difficult for drugs to reach the tumor site, thereby affecting therapeutic outcomes. Additionally, the rigid ECM not only restricts immune cells from entering the tumor but also exerts mechanical tension. This mechanical tension inadvertently stimulates mechano-transduction-mediated signaling, thereby interfering with the progress of immunotherapy (12). The stiff ECM also leads to hypoxia-induced interactions, which have been shown to impair the effectiveness of immunotherapy. Hypoxic environments make tumor cells more drug-resistant while also inhibiting the function of immune cells, further weakening the efficacy of immunotherapy (29). Therefore, fibrosis is not merely a localized tissue issue but a significant factor affecting the overall effectiveness of systemic immunotherapy.

This study downloaded three COAD-related datasets from the GEO database, namely GSE17536, GSE29621, and GSE74604, totaling 272 patient expression profile data. The CIBERSORT algorithm was employed to analyze cell subtypes in the COAD samples. It determined the abundance of various tumor-infiltrating immune cells (TIIC) types, including 22 types of cells. Among these, the feature data for WGCNA analysis consisted of the scores of 7 T cell subtypes. By employing WGCNA analysis, a gene co-expression network was built for COAD by utilizing the expression profiles of the leading 5000 genes. To explore the potential functions and mechanisms of CD8^+^ T cells, the black and turquoise modules, which have the highest association with CD8^+^ T cells, were selected for study. Following the utilization of the Metascape website for enrichment analysis, it was discovered that the genes within the black module exhibited significant enrichment in pathways related to membrane trafficking, protein localization, and metabolism. Conversely, the genes within the turquoise module demonstrated predominant enrichment in the extracellular matrix, vascular development, growth factor response, and similar categories. Four Hub genes, including *KCNE4*, *LARS2*, *SEZ6L2*, and *SOX7*, were selected through PPI analysis. Since the P-value of the *KCNE4* survival curve was greater than 0.05 and did not have statistical significance, no further research was conducted on it.

The infiltration of CD8^+^ T cells is associated with the prognosis of COAD patients. Our study revealed the difference in cell communications of CD8^+^ T cells between COAD and healthy samples according to scRNA-seq analysis. Then, we identified co-expressed modules associated with T cells (including CD8^+^ T cells) in the bulk RNA-seq dataset. Furthermore, we identified hub genes by random survival forest in the constructed PPI network. Our study revealed that the expression of identified hub genes was significantly associated with the prognosis of COAD patients, suggesting the importance of hub genes (log-rank test, P < 0.05). Moreover, the qRT-PCR for COAD cell lines and IHC experiment for 10 COAD tissues demonstrated the differential expression of hub genes between COAD and healthy samples. Differ from the currently essential genes in COAD, we, from the point of infiltration of CD8^+^ T cells in COAD, proposed and verified the hub genes (*SOX7*, *LARS2*, and *SEZ6L2*) in COAD by bioinformatics methods and experiments.

LARS2, one of the selected Hub genes, is predominantly found in mitochondria and plays a crucial role in translating mitochondrial genes, which includes the mitochondrial respiratory chain complexes I-V (30). Recent studies have found a close association between *LARS2* and the occurrence of nasopharyngeal carcinoma (31). *LARS2* can contribute to the progression of COAD by controlling leucine metabolism and tumor growth and influencing resistance to immunotherapy. Studies have shown that *LARS2* is highly expressed in LARS B cells, mediating the immune escape of COAD cells and promoting tumor progression. Additionally, other studies have discovered a notable association between *LARS2* and the clinical classification of COAD individuals, indicating its crucial involvement in the progression of COAD and facilitating the infiltration of CD4^+^ T cells (32). The experimental findings indicate that the expression level of *LARS2* in COAD samples is elevated compared to healthy samples. This observation was further confirmed through RT-qPCR and IHC techniques. Overexpression of *LARS2* can lead to mitochondrial dysfunction and increased levels of reactive oxygen species, causing renal epithelial cells to transition from a functional state to a fibrotic state, thereby promoting renal fibrosis (33). These findings help improve our understanding of the pathogenesis of COAD and provide a new perspective for predicting the response to immunotherapy. *LARS2* exhibited a significant positive correlation with CD8^+^ T cell infiltration. Given that *LARS2* is a mitochondrial leucyl-tRNA synthetase involved in mitochondrial protein synthesis and energy metabolism, its overexpression in COAD may contribute to an altered tumor metabolic environment, which in turn affects CD8^+^ T cell function. High levels of *LARS2* may be associated with immune evasion mechanisms, such as increased oxidative stress or lactate production, which can suppress CD8^+^ T cell cytotoxicity and hinder antitumor immune responses.

*SEZ6L2*, a protein similar to the Seizure-related 6 homolog in mice, is mainly found in the brain and belongs to the type 1 transmembrane protein family. *SEZ6* is a seizure-related gene 6 (*SEZ6*) family member, comprising *SEZ6L* and *SEZ6L2* (34). Notably, research has demonstrated that *SEZ6L2* cleavage can stimulate neurite outgrowth. Patients diagnosed with non-small cell lung cancer (NSCLC) who have tumors showing elevated *SEZ6L2* expression levels tend to experience reduced survival times specific to the tumor, in contrast to individuals without *SEZ6L2* expression (35). Research has additionally shown that *SEZ6L2* is elevated in COAD tissues and is associated with an unfavorable patient prognosis. Interestingly, *SEZ6L2* knock-out has been shown to promote apoptosis of COAD cells, affecting the ultimate size of xenografts in tumor models (36). High *SEZ6L2* expression has been detected in all examined CRC cell lines. Additionally, *SEZ6L2* sensitivity to cisplatin, dasatinib, and gemcitabine has been identified. The prognostic model shows that increased *SEZ6L2* expression is associated with reduced survival duration. Given these findings, *SEZ6L2* may serve as a prognostic biomarker for patients with COAD. No previous studies have elucidated the mechanism of the *SEZ6L2* in relation to fibrosis, underscoring the need for further research. *SEZ6L2* was found to be negatively correlated with CD8^+^ T cell infiltration. This suggests that *SEZ6L2* may play a role in immune suppression, possibly by modulating signaling pathways that promote T cell exhaustion or impairing antigen presentation. The correlation between *SEZ6L2* and reduced CD8^+^ T cell presence in the tumor microenvironment indicates that it may contribute to immune escape mechanisms, thereby facilitating tumor growth and resistance to immunotherapy.

*SOX7*, also known as the Sex-determining region Y-box7, belongs to the Sox gene family, which consists of eight subfamilies distinguished by the similarity of their HMG-box sequence (37). The *SOX7* is included in the F subfamily. It has been observed that certain members of the *SOX* gene family, including *SOX7*, exhibit reduced expression in COAD (38). This reduction is often governed by DNA methylation, and these genes tend to play a suppressive role in COAD. Research has shown that *SOX7* is expressed at diminished levels in COAD cells and tissues, and its expression is regulated by DNA methylation. In COAD cells, the restoration of *SOX7* expression can induce cell apoptosis, inhibit cell proliferation, and reduce clonogenic ability (39). Furthermore, the anti-cancer properties of *SOX7* have been discovered through its ability to suppress the Wnt/β-catenin signaling pathway (40). Our study identified *SOX7* as a hub gene in modules related to COAD and CD8^+^ T cell infiltration. We also found a similar correlation with the sensitivity to cisplatin, dasatinib, and gefitinib. Additional confirmation was obtained using real-time quantitative polymerase chain reaction (RT-qPCR), western blot, and IHC. This supported previous findings by demonstrating a decrease in the expression level of *SOX7* in COAD samples compared to healthy samples. However, this is the first instance where *SOX7* has been discussed in relation to CD8^+^ T cell infiltration in COAD. As for the role of SOX7 in influencing fibrosis formation, another study revealed a significant reduction in *SOX7* expression levels in both human and mouse fibrotic livers, with a pronounced decrease observed in fibrotic foci (41). Therefore, a comprehensive study of *SOX7* could elucidate the mechanisms underlying the occurrence and progression of gastrointestinal malignancies, providing new insights for biomolecular targeted therapies for these conditions. *SOX7* exhibited a positive correlation with CD8^+^ T cell infiltration. Given that SOX7 has been identified as a tumor suppressor that regulates Wnt/β-catenin signaling, its downregulation in COAD may lead to a more immunosuppressive tumor microenvironment. Since Wnt/β-catenin signaling has been implicated in preventing CD8^+^ T cell recruitment, the reduced expression of *SOX7* could contribute to the exclusion of cytotoxic T cells from the tumor, weakening immune-mediated tumor control.

As far as we know, this research is the initial examination of the association between *LARS2*, *SEZ6L2*, and *SOX7* levels and the infiltration of CD8^+^ T cells in COAD. However, there are some limitations to consider. Firstly, our study’s conclusions were drawn from public databases that were subject to data quality variations. Secondly, the biological functions of the three hub genes in COAD progression and tumor fibrotic process were not preliminarily demonstrated. Although the precise control of immune infiltration by *LARS2*, *SEZ6L2*, and *SOX7* in CD8^+^ T cells and tumor fibrotic process are not yet understood, the results are promising and justify additional research.

In conclusion, a three-gene predictive model was developed through a series of bioinformatics analyses to predict the outcomes of COAD patients. CD8^+^ T cells immune infiltration negatively correlated with the three hub genes, namely *LARS2*, *SEZ6L2*, and *SOX7*. As a result, we propose that *LARS2*, *SEZ6L2*, and *SOX7* could serve as novel prognostic biomarkers and potential immunotherapeutic targets in COAD.

## Data Availability

The datasets presented in this study can be found in online repositories. The names of the repository/repositories and accession number(s) can be found in the article/**Supplementary Material**.
