# Supplementary material for: Discovering biomarkers associated with infiltration of CD8+ T cells and tumor-associated fibrosis in colon adenocarcinoma using single-cell RNA sequencing and gene co-expression network
Source: Front Immunol. 2025 Mar 31;16:1496640. doi: 10.3389/fimmu.2025.1496640 (PMC11994618; doi:10.3389/fimmu.2025.1496640)
Supplement: Supplementary file 1 [file DataSheet1.docx]

**Discovering biomarkers associated with infiltration of CD8+ T Cells and tumor-associated fibrosis in colon adenocarcinoma using single-cell RNA sequencing and gene co-expression network**

Supplemental Figures


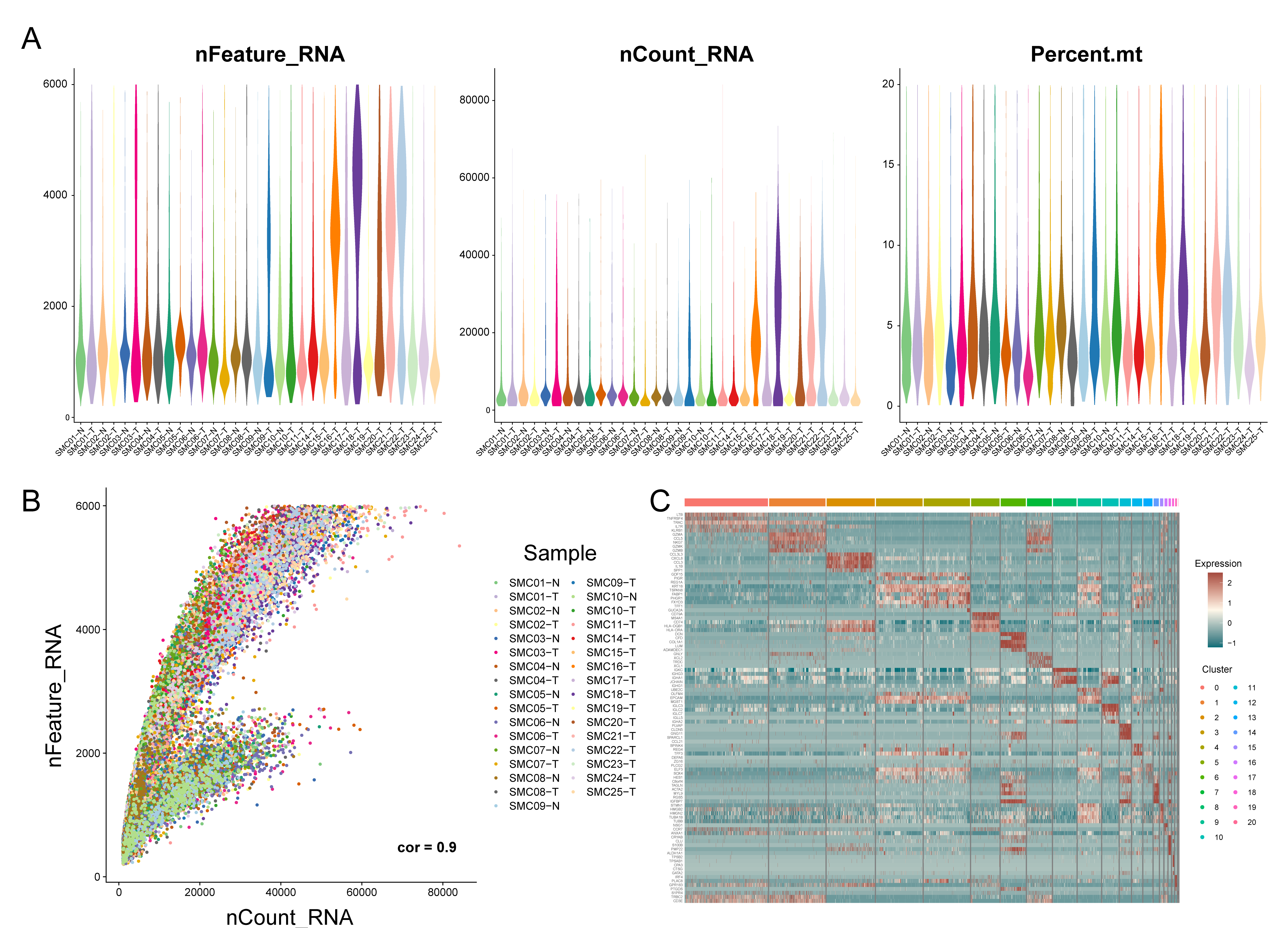


## Supplemental figure 1. QC of scRNA-seq data.

**(A)** Post-quality control filtering of each sequenced cell, which was plotted in violin plots to display their number of RNA features (nFeature_RNA) and absolute UMI counts (nCount_RNA). **(B)** Correlation analysis between nFeature and nCount. **(C)** Heatmap depicting expressions of top five marker genes among 21 detected CRC cell clusters.


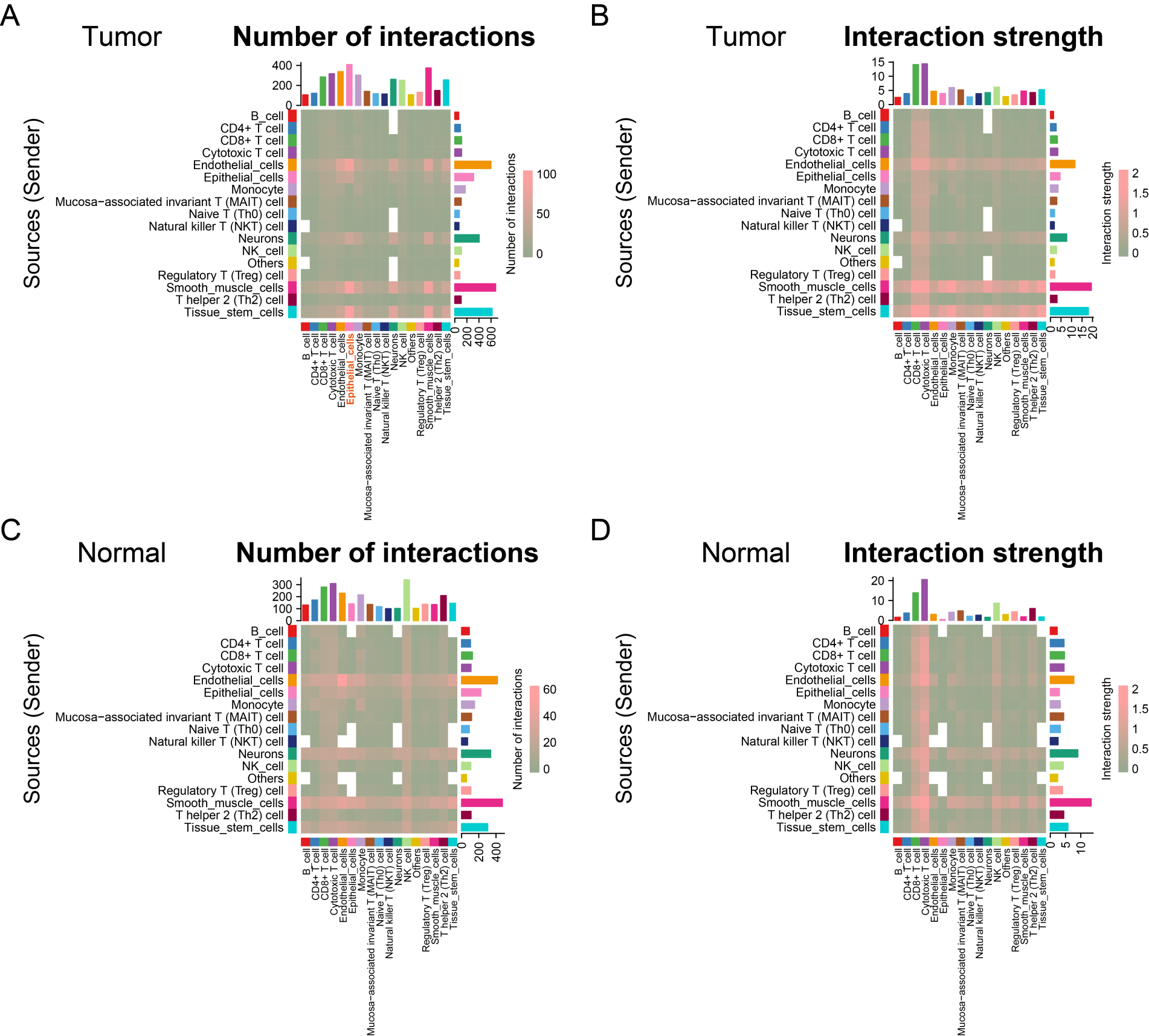


## Supplemental figure 2. Cell communications among cell types in tumor and normal samples*.*

**(A)**-**(B)** The number and strength of cellular communications among cell types in tumor samples. **(C)**-**(D)** The number and strength of cellular communications among cell types in normal samples.


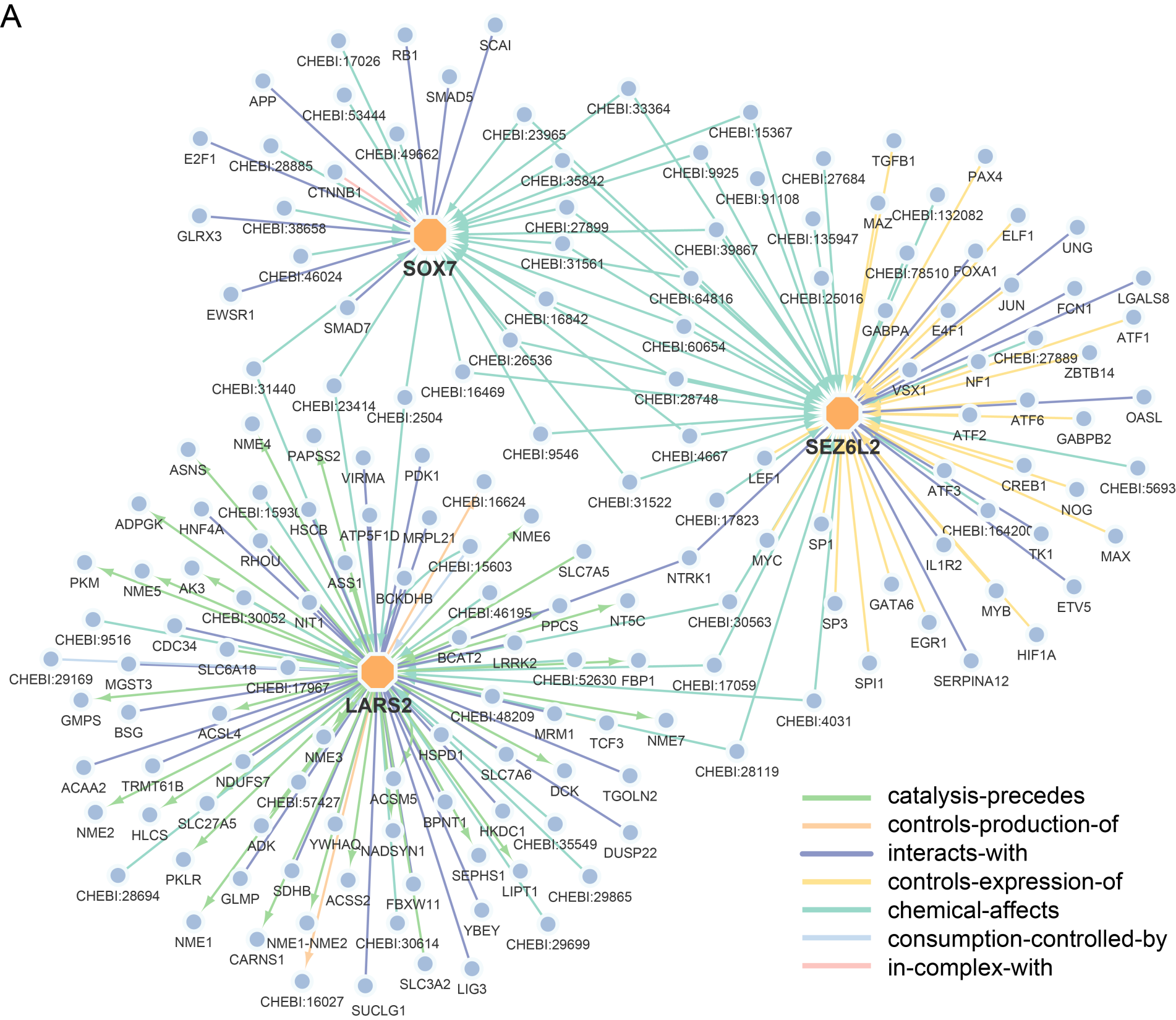


## Supplemental figure 3. Drug-gene and protein-protein interactions among hub genes and their partners.

(**A**) Drug-gene and protein-protein interactions of hub genes that were obtained from Pathway Commons database. The color of edges mean interacted types.


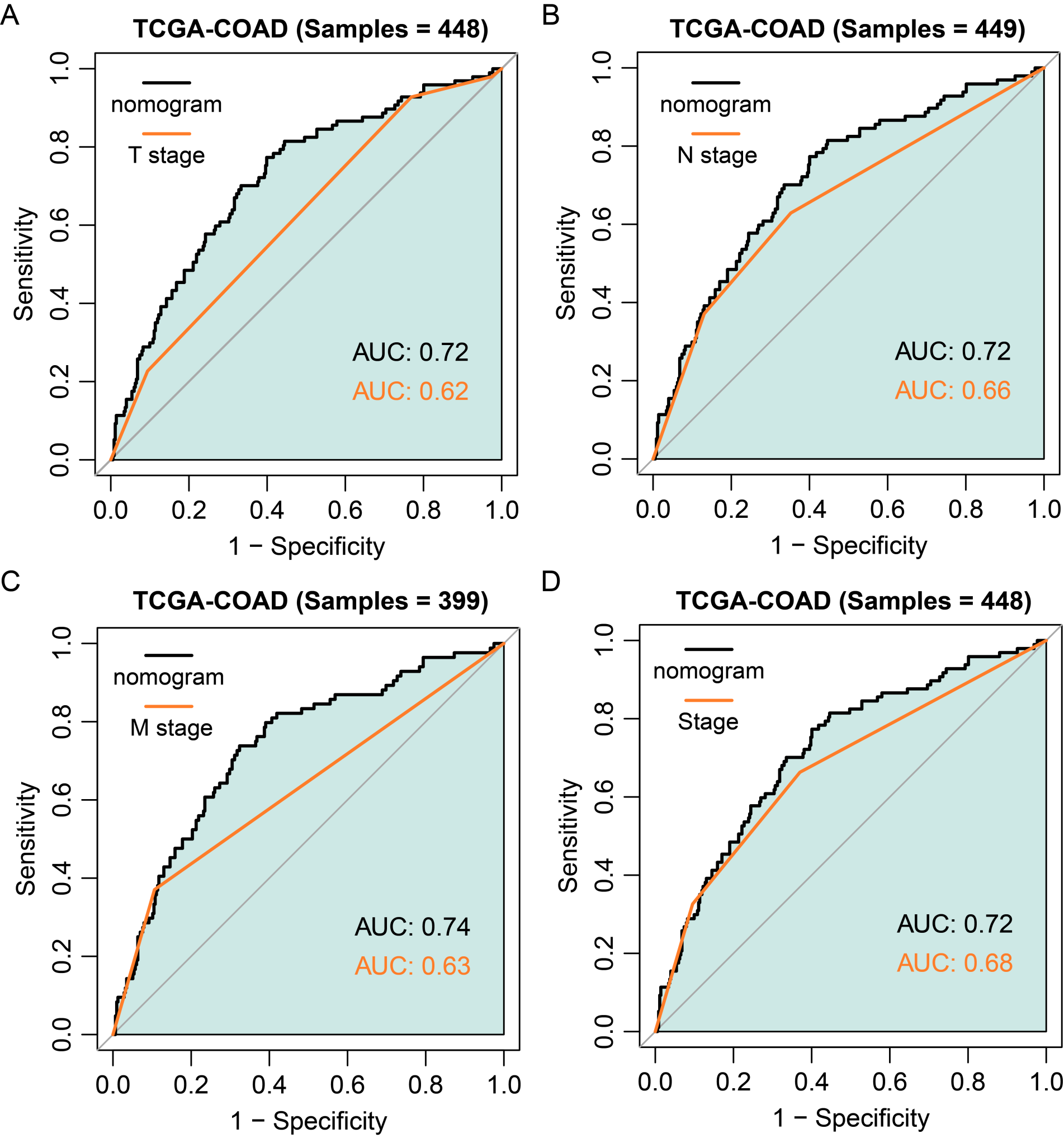


## Supplemental figure 4. Receiver operating characteristic (ROC) curve for nomogram and TNM stage system.

**(A)**-**(D)** ROC curve for nomogram and T stage, N stage, M stage, and Stage in TCGA dataset.


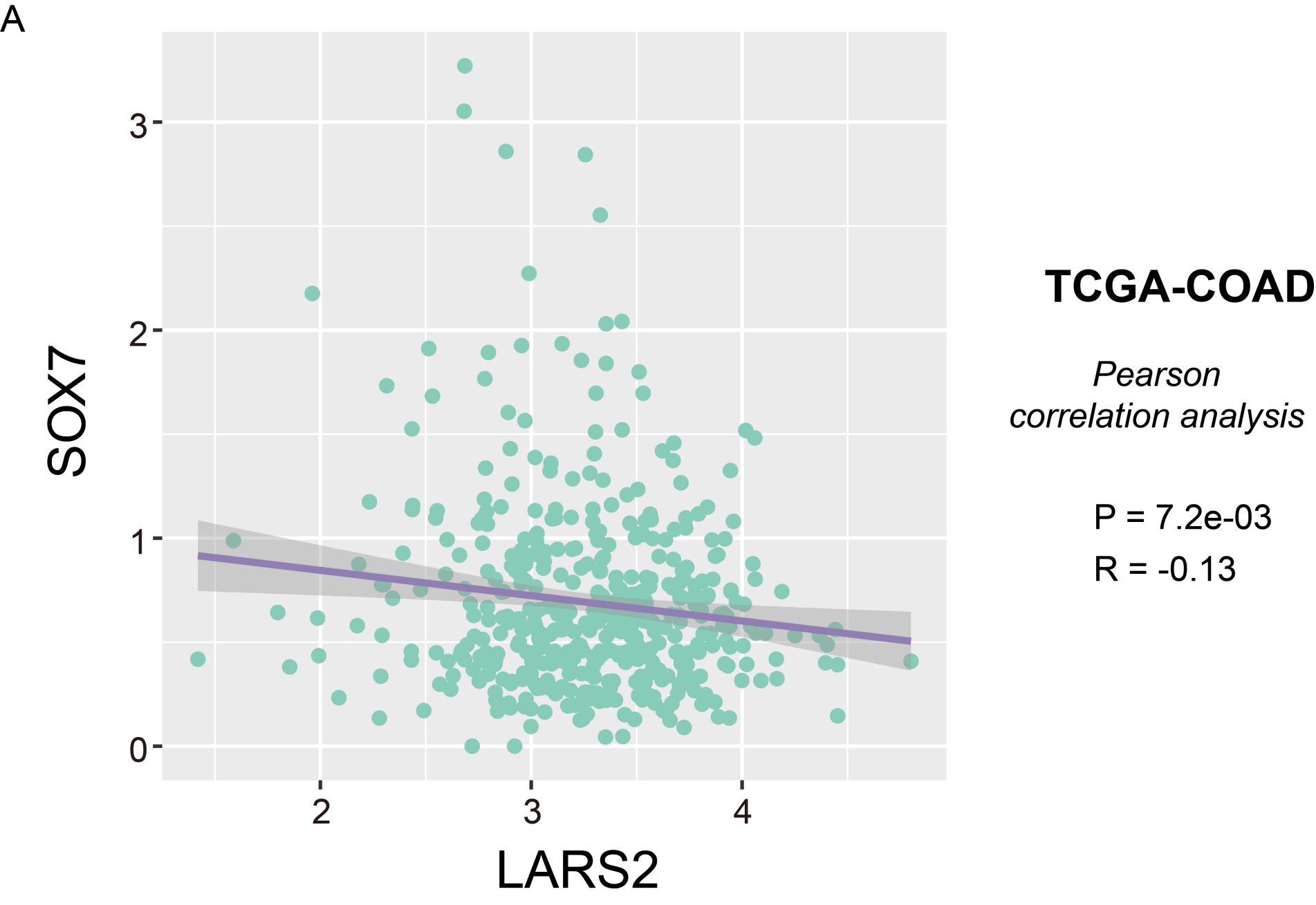


## Supplemental figure 5. Correlation between SOX7 and LARS2.

**(A)** The Pearson correlation between expression of SOX7 and LARS2 in TCGA COAD dataset.
