# Supplementary material for: Discovering biomarkers associated with infiltration of CD8+ T cells and tumor-associated fibrosis in colon adenocarcinoma using single-cell RNA sequencing and gene co-expression network
Source: Front Immunol. 2025 Mar 31;16:1496640. doi: 10.3389/fimmu.2025.1496640 (PMC11994618; doi:10.3389/fimmu.2025.1496640)
Supplement: Supplementary file 2 [file DataSheet2.doc]

Supplementary Information

**Supplementary Tables**

Table S1.Gene Co-expression Network

Table S2.Survival analysis of 3 genes
